# Actin cytoskeletal inhibitor 19,20-epoxycytochalasin Q sensitizes yeast cells lacking *ERG6* through actin-targeting and secondarily through disruption of lipid homeostasis

**DOI:** 10.1038/s41598-021-87342-4

**Published:** 2021-04-08

**Authors:** Kwanrutai Watchaputi, Pichayada Somboon, Nipatthra Phromma-in, Khanok Ratanakhanokchai, Nitnipa Soontorngun

**Affiliations:** 1 Division of Biochemical Technology, School of Bioresources and Technology, King Mongkut’s University of Technology Thonburi (KMUTT), Bangkok, 10150 Thailand; 2 Division of Fermentation Technology, Faculty of Food Industry, King Mongkut’s Institute of Technology Ladkrabang (KMITL), Bangkok, 10520 Thailand

**Keywords:** Biochemistry, Biotechnology, Cell biology, Chemical biology, Drug discovery, Genetics, Microbiology, Molecular biology

## Abstract

Repetitive uses of antifungals result in a worldwide crisis of drug resistance; therefore, natural fungicides with minimal side-effects are currently sought after. This study aimed to investigate antifungal property of 19, 20-epoxycytochalasin Q (ECQ), derived from medicinal mushroom *Xylaria* sp. BCC 1067 of tropical forests. In a model yeast *Saccharomyces cerevisiae*, ECQ is more toxic in the *erg6*∆ strain, which has previously been shown to allow higher uptake of many hydrophilic toxins. We selected one pathway to study the effects of ECQ at very high levels on transcription: the ergosterol biosynthesis pathway, which is unlikely to be the primary target of ECQ. Ergosterol serves many functions that cholesterol does in human cells. ECQ’s transcriptional effects were correlated with altered sterol and triacylglycerol levels. In the ECQ-treated Δ*erg6* strain, which presumably takes up far more ECQ than the wild-type strain, there was cell rupture. Increased actin aggregation and lipid droplets assembly were also found in the *erg6*∆ mutant. Thereby, ECQ is suggested to sensitize yeast cells lacking *ERG6* through actin-targeting and consequently but not primarily led to disruption of lipid homeostasis. Investigation of cytochalasins may provide valuable insight with potential biopharmaceutical applications in treatments of fungal infection, cancer or metabolic disorder.

## Introduction

The incidence of the COVID-19 pandemic provides a valuable lesson and increases our awareness on dangerous virus as well as other bugs, including microbes. Microbial drug-resistance is indeed a real threat to the global community and economy. International agencies and experts have predicted that there could be over 10 million deaths yearly by the year 2050, and, ten years from now, antimicrobial resistance could force up to 24 million people at risk of extreme poverty (World Health Organization). The need for prevention and efforts to overcome antimicrobial resistance are now urgently called for. On a rise, invasive fungal infections have caused high morbidity and mortality worldwide, especially in immunocompromised people including AIDS (acquired immune deficiency syndrome) and cancer patients as well as transplant recipients, and even children and senior citizen are also at risk. Mortality rates in critically ill patients are high, ranging from 40 to 70%, depending on the severity of the infections^1^, while few antifungals are available for the treatment of fungal infections. For the past 60 years, among the antifungals available, amphotericin B (AmB) has been developed and used as the gold-standard antifungal for treatment of invasive pathogenic fungal infections^2^. AmB targets ergosterol, the main sterol component of the fungal cell membrane, possibly through generation of membrane pores that cause leakage of ions and subsequent fungal cell death. However, the administration of AmB is limited because it also interacts with the cholesterol present in human cell membranes and causes severe undesired side effects^3^. The azole antifungals developed in the early 1980s target enzymes of the ergosterol biosynthesis pathways and display lower toxicity^4,5^. Azoles are therefore commonly used in clinical practice despite their fungistatic effect which eventually leads to the development of drug resistance. Alternatively, lower administered doses of AmB in combination with azoles or other classes of antifungals are often recommended in current antifungal therapies^6^.

The primary mechanisms of action of most antifungal drugs is to disrupt the cellular composition of the fungus and to alter the function of membrane-associated proteins, compromising the integrity of the fungal membrane^7^. Since the yeast ergosterol and human cholesterol are structurally and functionally similar, the occurrence of multiple adverse reactions may be observed^3^. Biosynthesis of sterols in yeast is a multistep control process. An early, key step is the rate-limiting conversion of 3-hydroxy-3-methylglutaryl coenzyme A (HMG-CoA) to mevalonic acid catalyzed by HMG-CoA reductase, encoded by the *HMG1* and *HMG2*^8^*.* These two isoenzymes display different modes of feedback control and regulation^8^. The accumulation of squalene in *HMG1*-overexpressing strains suggests additional bottlenecks in the postsqualene formation steps^9,10^. In fact, there are overlapping mechanisms for controlling ergosterol biosynthesis. These include transcriptional regulation by the sterol regulatory element transcription factors Upc2 and Emc22, the heme-binding regulator Hap1 and the repressors Rox1 and Mot3 as well as a feedback inhibition of enzymes and changes in their subcellular localization^11^. Enhanced squalene accumulation is shown in a mutant overexpressing mutant Hmg2; however, additional deletion of *ERG6* do not further enhance squalene accumulation but transfer surplus squalene into C27 sterols, suggesting a lack of ergosterol feedback inhibition^12^. The synthesis of ergosterol in yeasts is a complex pathway, involving more than 20 distinct reactions. The sterol biosynthesis pathways of ergosterol and cholesterol are highly similar, except for the final steps of the pathways^13^. Disruption of *ERG* genes in the late step of the ergosterol biosynthesis pathway also results in lower activity of Pdr5p drug efflux transporter and susceptibility to stresses^14^.

Due to the limited availability of antifungal agents for combating an increasing number of fungal infections, the urgent need for new antifungal drugs remains an important public health issue worldwide^15^. Novel antifungals with effective fungicidal activity and specificity for fungal targets are important to fight against drug-resistant fungi. In nature, a variety of filamentous fungi produce bioactive compounds for self-defence, thereby offering potential sources of valuable drug leads, including caspofungin (a semisynthetic compound modified from fungi-derived pneumocandin B0), penicillin, and griseofulvin^16^. In recent years, a plethora of new bioactive secondary metabolites of interest and potential drug leads are derived from filamentous fungi. *Xylaria* spp., present in tropical forests, have also been reported for application in traditional Chinese folk medicines with potential biological properties such as immune-modulatory, antimalarial, antifungal, antiviral, and anticancer properties^7^. For examples, *Xylaria primorskensis* produces xylaranic acid with potent antibacterial activity^17–19^ or, *Xylaria sp. Acra L38* extract displays antifungal activity and contains medicinal agents such as zofimarin^20^ and griseofulvin, which inhibit fungal plant pathogens^21^. Despite its high potential, there has been little exploration of biopharmaceutical applications for *Xylaria* spp. Recently, a cultural extract of the fungal *Xylaria* sp. BCC 1067 was reported to be a potential source of novel antifungal agents^22^. This study aimed to further characterize the antifungal action of 19,20-epoxycytochalasin Q (ECQ), present in the extract of *Xylaria sp.* BCC 1067^23^, in mediating fungal cell death and lipid homeostasis. An alteration in sterol biosynthesis following ECQ treatment was investigated using *S. cerevisiae* wild-type and the Δ*erg6* strains, which the latter lacks a key antifungal target enzyme of ergosterol biosynthesis, as a model for study.

## Results

### Antifungal activity of 19,20-epoxycytochalasin Q against the *S. cerevisiae ERG* deletion strains

**Figure 1:**
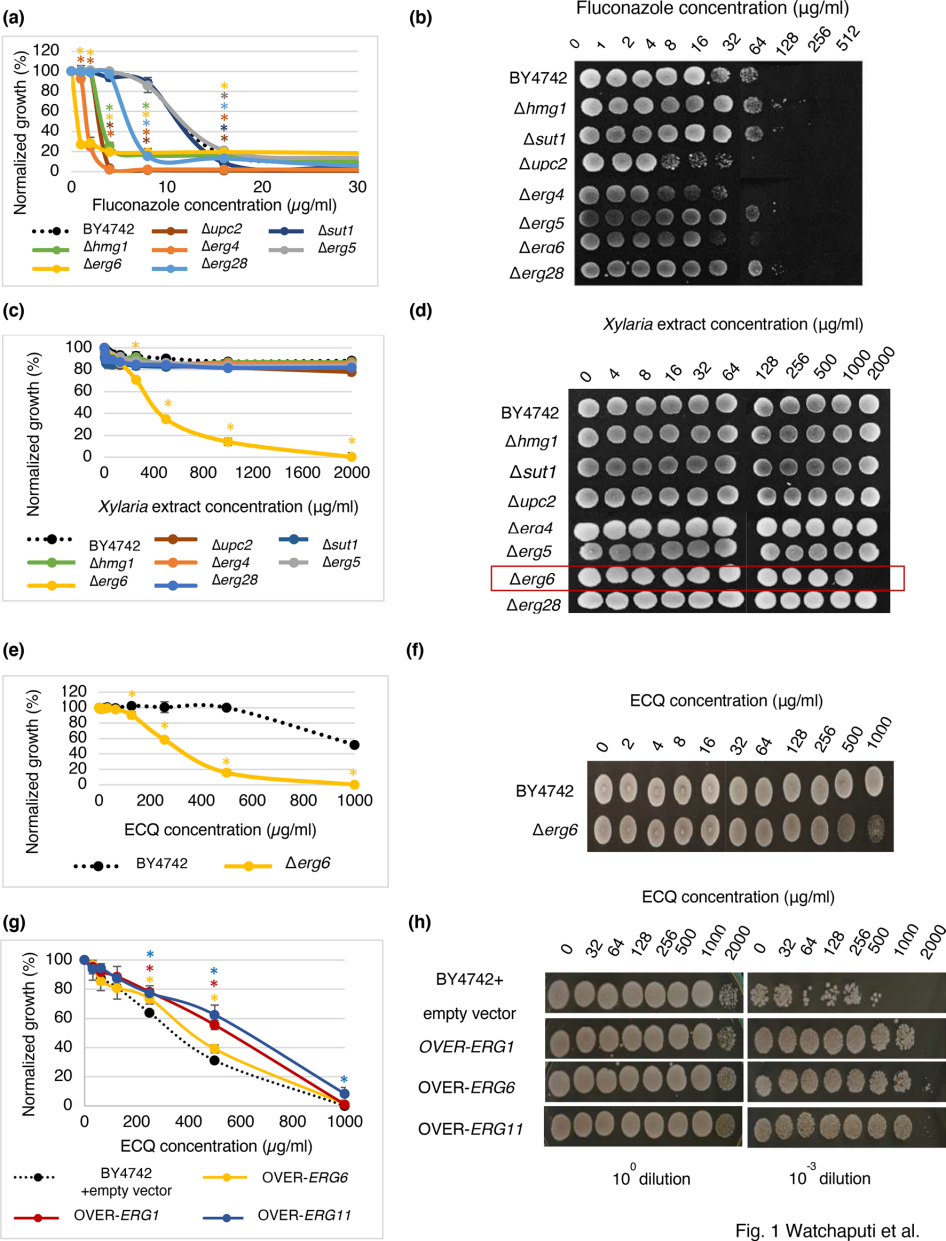
Susceptibility and survival of *S. cerevisiae* strains with a deletion or overexpression in the gene for the ergosterol biosynthesis during the treatments with different concentrations of fluconazole (**a**, **b**), *Xylaria* extract (**c**, **d**), or ECQ (**e**–**h**) using micro-dilution assays and spot tests, respectively. Cells from micro-dilution assays were directly spotted (10^0^ dilution) or diluted 1000 times (10^–3^ dilution) prior to be spotted on YPD plates. Growth of the overexpression strains were compared with the wild-type strain. * was referred to the mean difference with significant *p* value of < 0.05.

Identification of bioactive compounds present in the cultural extract of *Xylaria* BCC 1067 indicated that 19,20-epoxycytochalasin Q (ECQ) is present, in addition to buformin, α-peltatin, 3-methylhistidine and some acid derivatives such as gluconic acid, 4-guanidinobutanoic acid, gibberellic acid, and picolinic acid. ECQ possess interesting biological activities, including an antibacterial and antiplasmodial property^24^; therefore, it was selected for further investigation. To examine the antifungal action of ECQ, the susceptibility of mutant yeast strains with a deleted gene of the enzyme in the ergosterol biosynthetic pathway was first investigated since many of them are served as antifungal drug targets. The extract of *Xylaria* sp. BCC 1067 at concentrations ranging from 0 to 2000 µg/ml and a positive control antifungal drug fluconazole, known to target the Erg11p enzyme, were also included. In comparison to the parental BY4742 strain, the Δ*upc2*, Δ*erg4,* and Δ*erg6* strains showed increased sensitivity to fluconazole, as shown by growth assays and serial dilution spot tests (Fig. 1a,b) as previously reported^25^. Growth of the Δ*hmg1* and Δ*erg28* strains was slightly inhibited but not that of the Δ*sut1* and Δ*erg5* strains (Fig. 1a). Interestingly, only the Δ*erg6* strain had increased sensitivity to the *Xylaria* extract tested when compared to the wild-type *S. cerevisiae* strain, with exhibited MIC_50_, MIC_80_ and MFC values of 375 µg/ml, 900 µg/ml and 2,000 µg/ml, respectively (Fig. 1c,d). The involvement of Erg6p in conferring resistance to the *Xylaria* extract was then further investigated using the purified ECQ. Selectively, the **∆erg6** strain exhibited increased sensitivity to ECQ, with an MIC_50_ value of 295 µg/ml and an MFC value of 1000 µg/ml (Fig. 1e), which displays slightly better antifungal activity to that observed from the extract of *Xylaria*. At 1000 µg/ml of ECQ, the ∆*erg6* strain could barely survive, indicating the important role of Erg6p in mediating resistance to antifungal ECQ (Fig. 1f). Additional antifungal assays indicated that *ERG6-* , *ERG1*- or *ERG11*-overexpressing strains show better tolerance to ECQ as compared to the parental strain (Fig. 1g,h). This could be because these *ERG*-overexpressing strains produce enough or high ergosterol content for maintaining plasma membrane integrity; therefore, they showed ECQ resistance. However, the maximum growth and viability also depended on the level of expression of each gene contained in the plasmid. *ERG11* confered the highest ECQ resistance when cell viability was compared at an equal level of gene expression. Here, *ERG1* expression level was approximately 40X higher than those of *ERG11* and *ERG6* genes.

### ECQ induced the expression of the fungal-specific *ERG6* gene

**Figure 2:**
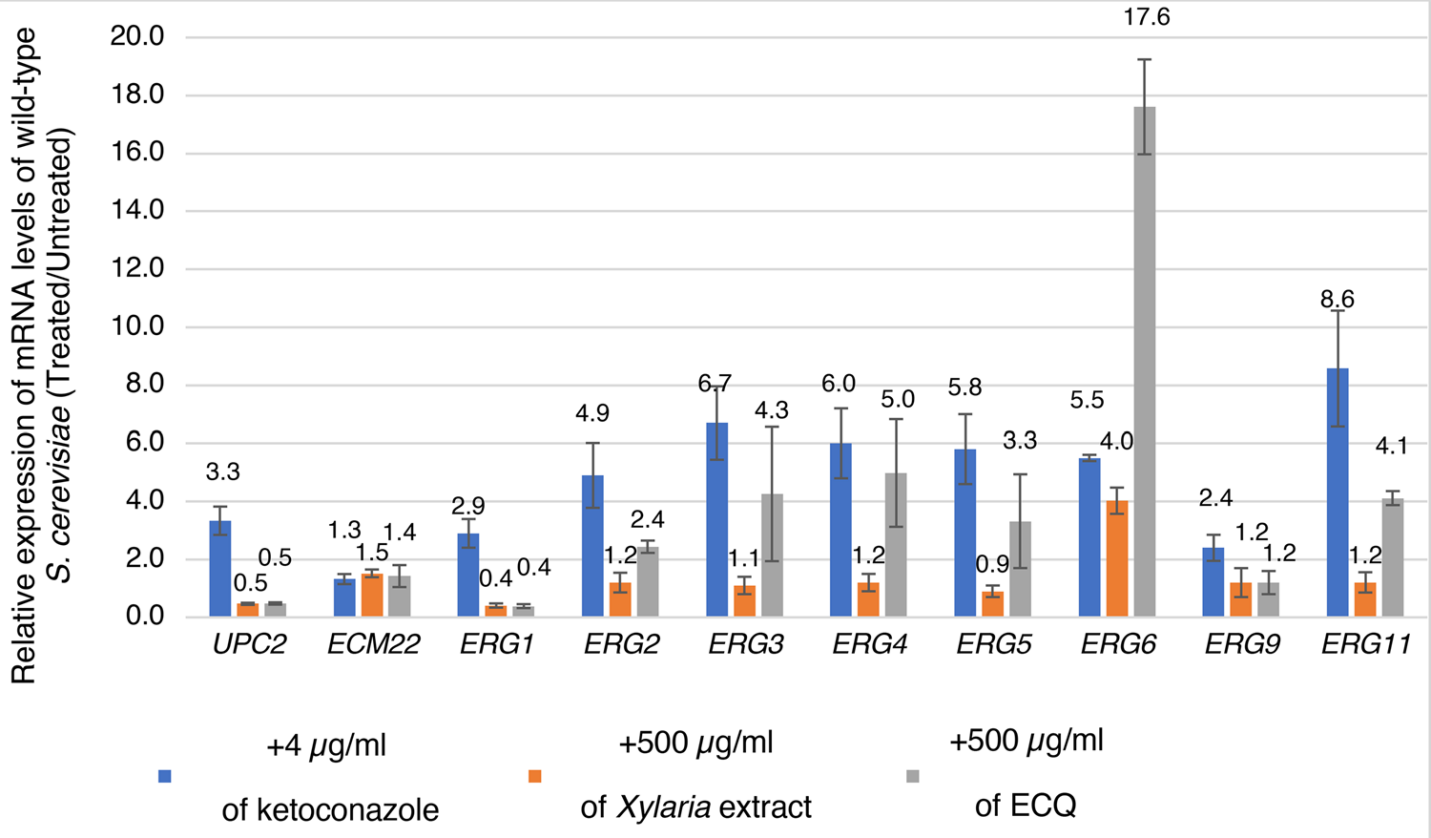
Relative expression of genes in ergosterol biosynthesis. *S. cerevisiae* BY4742 wild-type strain was treated with 4 µg/ml of ketoconazole, 500 µg/ml of *Xylaria* extract, or 500 µg/ml ECQ for 2 h. The relative mRNA levels of the treated cells were compared to the untreated cells and normalized with a housekeeping gene *TDH3*. At least two independent qRT-PCR experiments were performed in triplicates. Error bars represent standard error of the mean (SEM).

Previous work has shown that antifungal drugs including ketoconazole induce the expression of many genes, including ergosterol biosynthetic genes, required for conferring drug resistance^26–28^. The qRT-PCR analysis was therefore performed by using *TDH3* as a house-keeping gene, and the results confirmed that ketoconazole induces the expression of genes in the ergosterol biosynthetic pathway (*ERG11*, *ERG2*, *ERG3*, *ERG4*, *ERG5*, and *ERG6*) as well as transcription of the regulatory gene *UPC2* involved in the up-regulation of these *ERG* genes (Fig. 2a). Interestingly, the expression of the *ERG6* gene was strongly induced by 4.0-fold and 17.6-fold after treatment with 500 µg/ml of *Xylaria* extract or ECQ, respectively (Fig. 2a). The expression of additional *ERG* genes, namely *ERG11*, *ERG5*, *ERG4, ERG3*, and *ERG2*, in late ergosterol biosynthesis was also induced by ECQ by 4.1-, 3.3-, 5.0-, 4.3- and 2.4-fold, respectively, when compared to the untreated cells (Fig. 2a). Expression of *ERG1* gene was decreased by 2.5- after induced with *Xylaria* extract or ECQ (Fig. 2a). Moreover, the expression of *UPC2* was also decreased by 2.0-fold (Fig. 2a) suggested up-regulation of genes in the biosynthesis of ergosterol but not of other pathways under the control of transcription factor Upc2p. Perhaps, ECQ may alter the levels of sterols, such as ergosterol, through alteration of gene expression as a potential feedback mechanism, similar to what has been previously observed for some antifungals^28,29^.

Unusually, the *Xylaria* extract could not induce expression of other *ERG* genes except for *ERG6*, suggesting that *ERG6* expression may alternately be in response to impurities (Fig. 2). However, ratio of ECQ in crude *Xylaria* extract was investigated the by HPLC and found that ECQ appeared approximately 27% in the extract (Supplementary Fig. 1) which may not contain enough ECQ to induce expression of other *ERG* genes. It remains to be shown. Nevertheless, further investigation of the role of the *ERG6* gene, encoding delta (24)-sterol C-methyltransferase for the conversion of zymosterol to fecosterol of the ergosterol biosynthetic pathway, was initiated. Erg6 is also important for the resistance of cells to antifungal azoles and echinocandins as well as tolerance to acid stress^30^.

### ECQ disrupted cellular actin organization and membrane integrity

**Figure 3:**
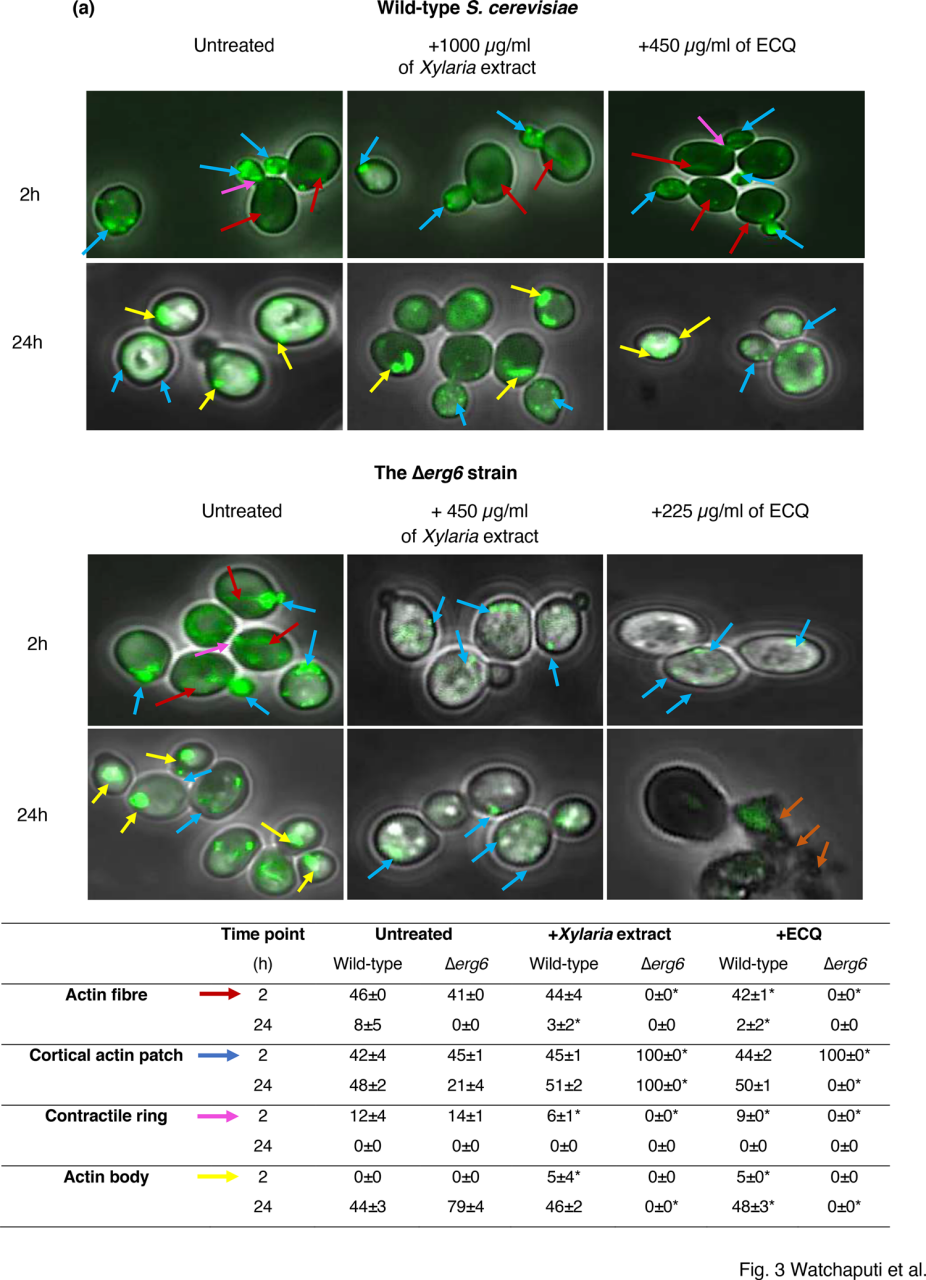

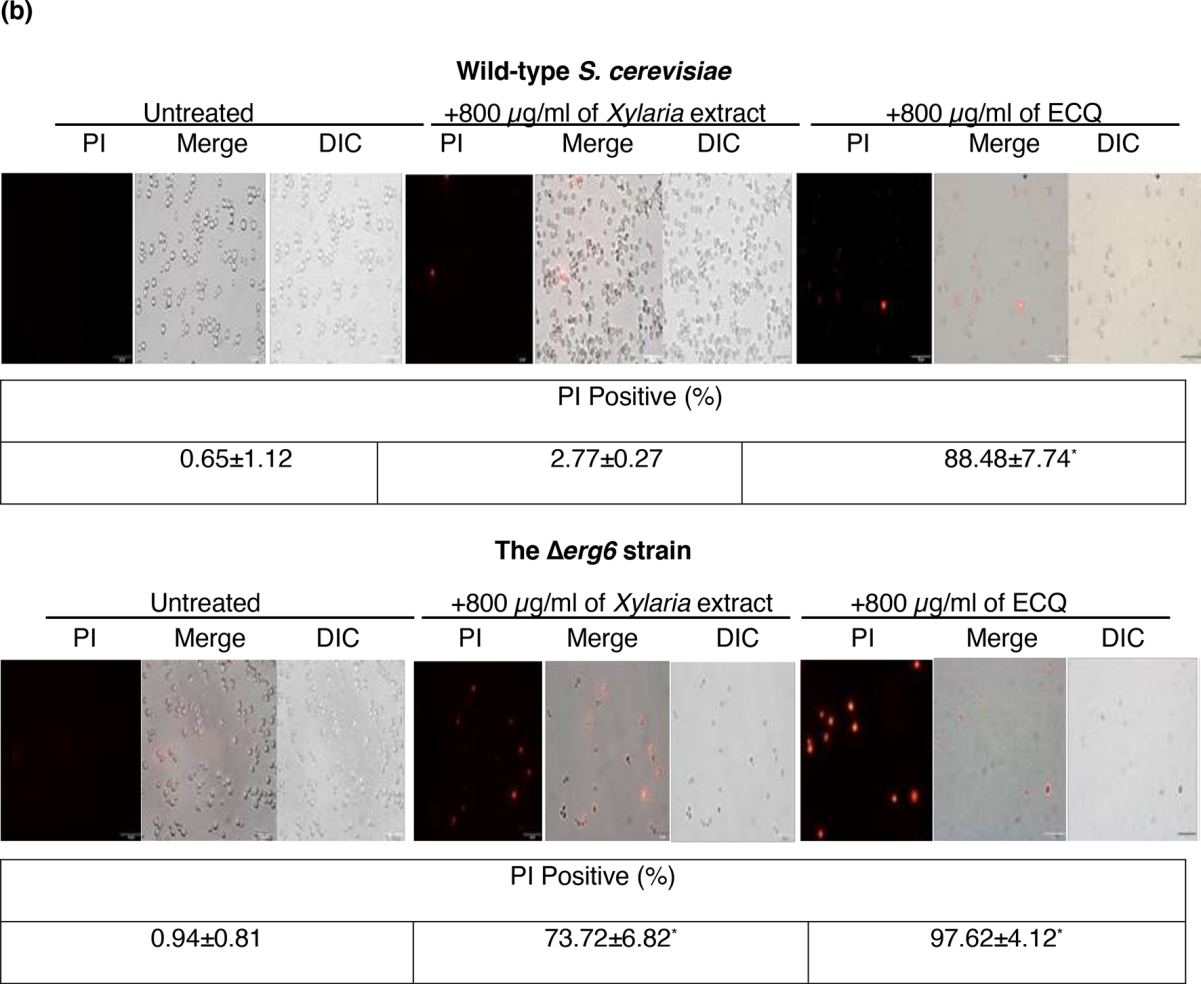
Effect of *Xylaria* extract and ECQ on actin cytoskeleton organization (**a**) and cell membrane integrity (**b**) in the wild-type *S. cerevisiae* and the ∆*erg6* strains. Blue arrows indicated cortical actin patches; red arrows indicated actin fibre; yellow arrows indicated actin body; and orange arrows indicated cell breakage. Error bars represent standard error of the mean (SEM) (*p < 0.05, using one-way ANOVA compared to the untreated condition).

Cytochalasins are natural bioactive compounds that have been extensively studied. They are known to disrupt actin organization, which has an important role in a variety of cellular processes. During polarized growth, yeast cells contain two types of actin structures which include actin fibre and cortical actin patches. The actin structures play an important role for intracellular transport. Actin fibre acts in the transport of secretory vesicles, whereas actin patches act as ports for the recycle of membrane components from the cortex^31^. Moreover, actin bodies which are F-actin aggregates are observed in stationary-phase cells and inferred to be markers of quiescence^32^. To date, cytochalasins B, D, F, and H have been reported in ability to disrupt actin^33^. The role of cytochalasin Q, namely ECQ to disrupt the organization of the actin n was recently shown using mutant *S. cerevisiae pdr5* and *Candida cdr1* strains^23^. Here, the actin organization of Δ*erg6* cells with compromised ergosterol biosynthesis was investigated, using an actin-specific phalloidin-FITC labelled stain^34^. The wild-type *S. cerevisiae* cells were incubated with concentrations of 1000 µg/ml of the *Xylaria* extract or 450 µg/ml of ECQ, while the ∆*erg6* cells were incubated with concentrations 450 µg/ml or 225 µg/ml, respectively. The results showed that two types of actin structures, including cortical actin patches (blue arrow) and actin fibre (red arrow), formed at the bud site and within the cell, indicating normal actin organization in the untreated wild-type and the ∆*erg6* strains at 2 h (Fig. 3a,b). At 24 h, yeast cells were going to the stationary phase, leading F-actin to aggregate and to form actin body structure (yellow arrow) as shown in Fig. 3a,b. After *Xylaria* extract treatment, no apparent change in actin fibre formation was observed in the wild-type strain, while the actin fibre disappeared in the *∆erg6* strain at all indicated time points, suggesting disruption of actin polymerization in the cytosol of the *∆erg6* strain (Fig. 3a,b). For treatment with ECQ, disruption of actin organization was observed after treatment at 24 h in the wild-type and as early as 2 h in the ∆*erg6* strain (Fig. 3a,b). ECQ disrupted organization of actin and, this effect of actin aggregation was more obvious in the *erg6*∆ strain that likely takes up more drug and more prone to ECQ toxicity.

Moreover, cell membrane breakage was observed in the ∆*erg6* strain after treatment with ECQ. As shown, treatment with ECQ at 24 h resulted in damaged cell membranes, membrane rupture, and possibly leakage of the cellular contents of the ∆*erg6* strain (Fig. 3b). The possibility that ECQ may disrupt plasma membrane integrity was tested. Polar fluorescent propidium iodide (PI) stain (MW = 668.4 Da), which binds to nucleic acids but does not permeate through the membrane, was used to score the number of cells in a population with lost cellular integrity^35^. The untreated *S. cerevisiae* wild-type and the Δ*erg6* strains exhibited a non-PI-positive signal with less than 1.0% (Fig. 3a,b). However, the *Xylaria* extract or the ECQ-treated wild-type strain and especially the Δ*erg6* strain exhibited a strong red fluorescent signal, positive for PI staining, which accounted for 2.77% and 88.48% of total cells for the wild-type and 73.72% and 97.62% of total cells for the Δ*erg6* strain, respectively (Fig. 3a,b). Overall, ECQ interferes with actin organization and causes defective cell morphology, resulting in plasma membrane breakage and cell death.

### *ERG6* deletion or ECQ treatment altered the sterol composition of yeast cells

**Figure 4:**
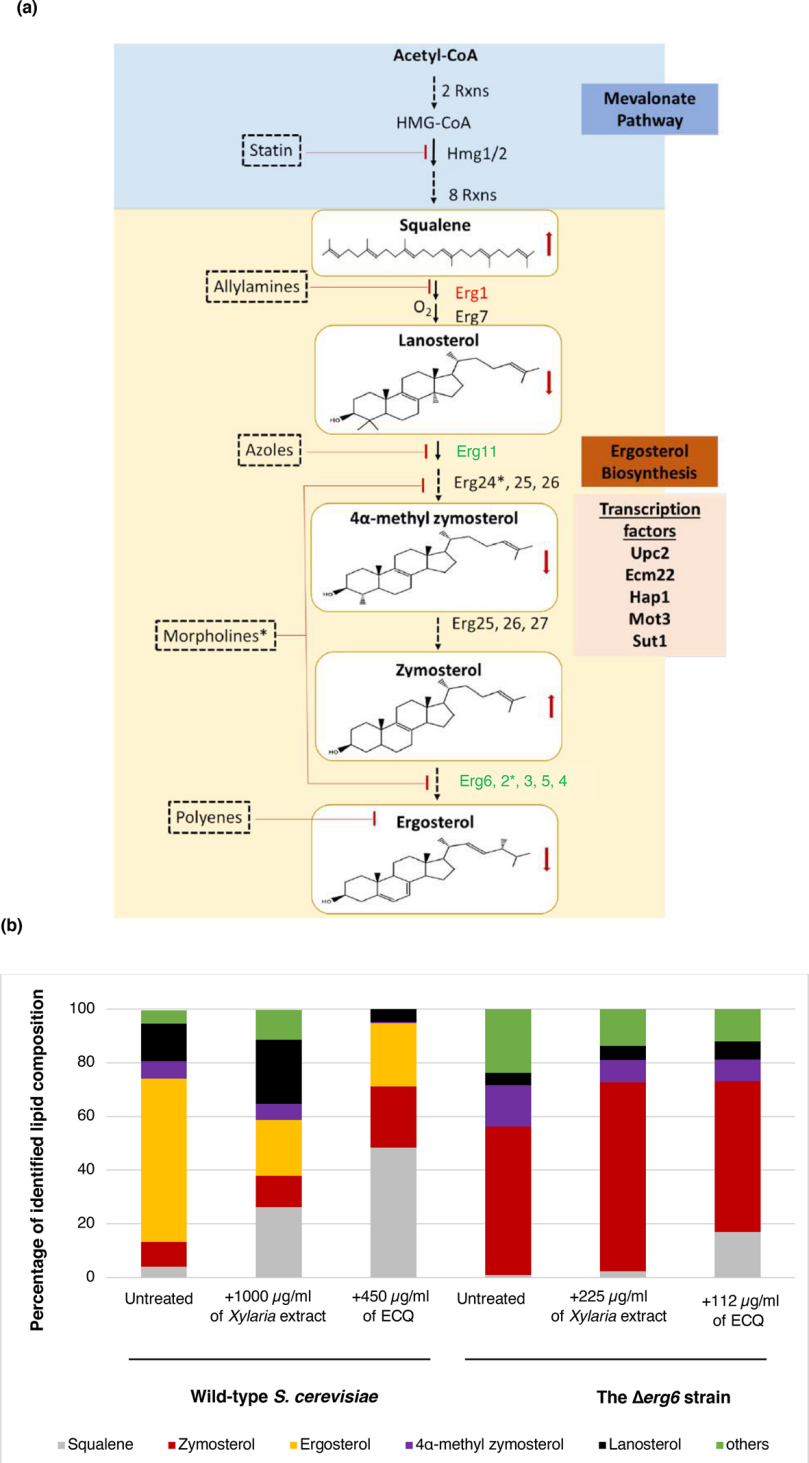

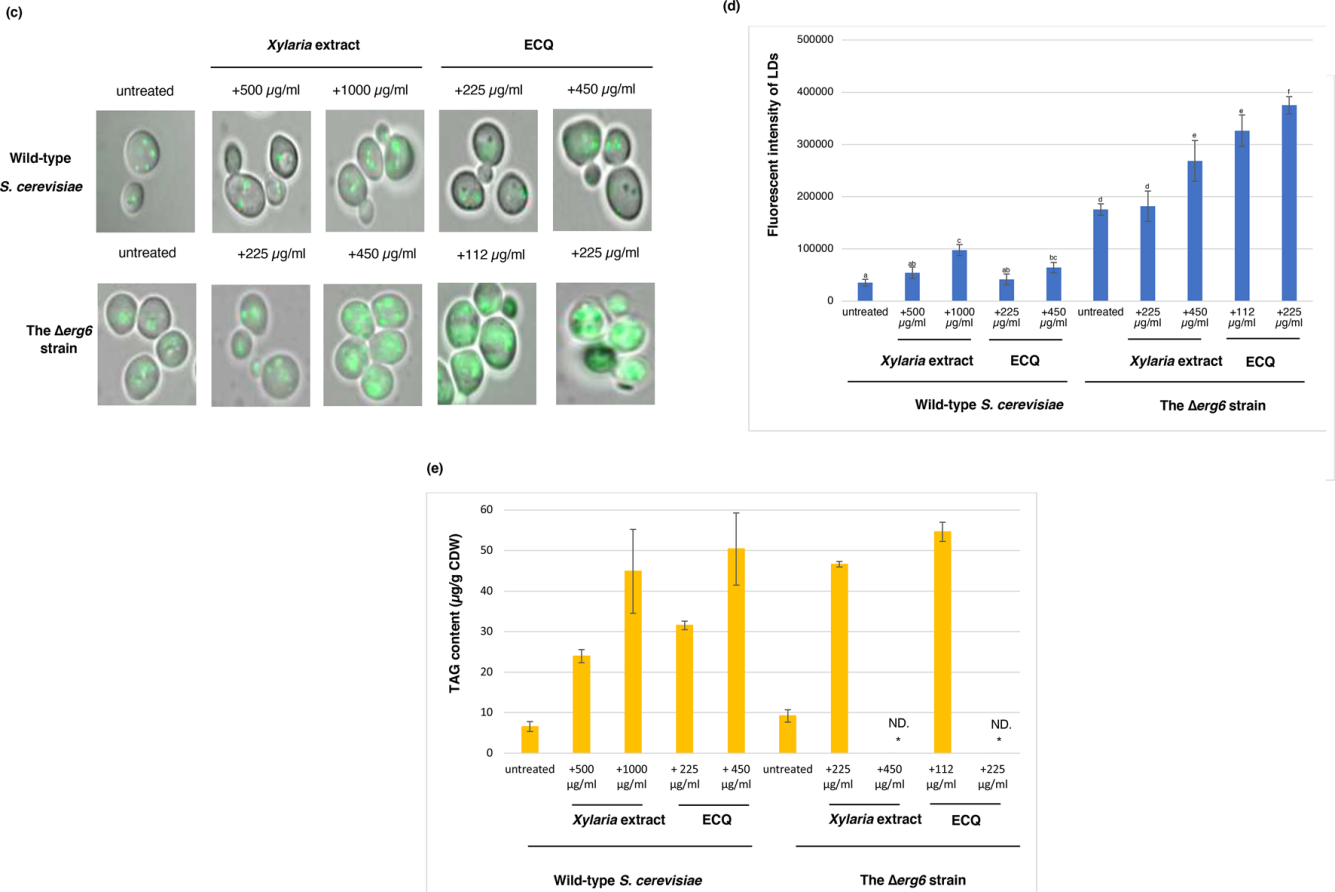
Alteration of sterol and TAG levels as well as formation of LDs clustering were examined during the ECQ treatment in *S. cerevisiae*. (**a**) ergosterol biosynthesis pathway in *S. cerevisiae*, including involved transcriptional factors and targets of antifungal drugs was depicted (**b**) percentage of identified sterol composition as quantified by GC–MS, (**c**) LDs formation and Nile Red fluorescent intensity, and the chromatograms of the change in (**d**) sterol composition and (**e**) TAG content of yeast cells after treatment with ECQ of *S. cerevisiae* wild-type and ∆*erg6* strains treated with the *Xylaria* extract or ECQ. Gene labelled in green or red colour indicated up- or down-regulated expression, respectively. Asterisks indicated target of Morpholines. Red arrows indicated increased or decreased accumulation of sterol level, following the ECQ treatment. “ND.” Was referred to “not detected”. Error bars represent standard error of the mean (SEM). Different letters above the error bars (**a**–**f**) indicate significant differences at *p* value of < 0.05.

A diagram represented the mevalonic pathway and the ergosterol biosynthesis was depicted to include antifungal drug key target enzymes, sterols and responsible transcription factors (Fig. 4a). In yeast, the early step is initiated by acetyl-CoA to produce an important intermediate, farnesyl pyrophosphate (FPP)^36^. Therefore, mutations during this step of the pathway are lethal. The late step of ergosterol biosynthesis is dedicated to the conversion of FPP to the sterol precursor squalene, the first specific intermediate for the production of ergosterol, mediated by Erg9p. The next step is conversion of squalene to lanosterol, the first sterol molecule of the ergosterol biosynthetic pathway that is mediated by the enzymes Erg1p (squalene epoxidase) and Erg7p (lanosterol synthase)^14^. Conversion of lanosterol to ergosterol requires many enzymes, including Erg11p and Erg6p^37^. Deletion of genes in ergosterol biosynthesis alters the sterol and lipid composition of the yeast cell membrane^38^. As shown, the Δ*erg6* strain is unable to catalyse C-24 methylation, leading to accumulated zymosterol^39^. Lacking of ergosterol biosynthetic enzymes support a critical role for ergosterol in yeasts and fungi^40^. In addition, when Erg11p is inhibited, a pathway for alternative sterol biosynthesis that leads to 14 methylergosta 28-24-28 dienol, is activated by treatment with antifungal azoles^41^. Likewise, treatment with ECQ induced cellular feedback through reprogramming the expression of many *ERG* genes, particularly *ERG6* (Fig. 2). Consequently, the alteration of sterol composition in the membrane may be resulted. Using gas chromatography-mass spectrometry (GC–MS), the sterol composition of the wild-type and the *erg6*Δ strains treated with ECQ was examined.

The GC–MS results showed that ergosterol is the main sterol component of the wild-type *S. cerevisiae* cell membrane in the untreated cells, along with the presence of squalene, zymosterol, 4α-methyl zymosterol, lanosterol and others (Fig. 4b). Treatment with the *Xylaria* extract disrupted normal ergosterol biosynthesis as shown by reduced ergosterol content of more than 60% of the total sterol fraction and increased accumulation squalene, lanosterol, zymosterol and others (Fig. 4b). In the wild-type strain under ECQ treatment, the ergosterol level was also reduced by approximately 60% compared to the total sterol fraction of the untreated cells (Fig. 4b). ECQ’s transcriptional effects on this ERG pathway were matched by alterations to levels of sterol and of triacylglycerol, but it is not possible to determine if the primary effect on lipid levels is transcriptional or not.

Remarkably, over an 1100% increase in squalene of the total sterol fraction was observed (Fig. 4b). Squalene has been reported to be a toxic lipid species in yeast; excess squalene is normally stored in LDs to prevent its toxicity^42,43^. In fact, the wild-type cells remain viable even with elevated squalene, suggesting normal squalene storage. Regarding the Δ*erg6* strain, an alteration in the sterol profile was dramatically observed (Fig. 4b). Strikingly, the ergosterol level was undetected, but a high accumulation of zymosterol and 4α-methyl zymosterol was observed, in comparison to the total sterol fraction of this deletion strain (Fig. 4b). When compared to untreated condition, treatment with the *Xylaria* extract or ECQ resulted in increasing levels of zymosterol by 27% and 2% of the total sterol fraction of the Δ*erg6* strain (Fig. 4b), respectively. Level of toxic squalene in the untreated Δ*erg6* strain increased by 100% and 600% of the total sterol fraction after treatment with the *Xylaria* extract or ECQ, respectively, thereby explaining increased sensitivity of the ECQ-treated Δ*erg6* strain (Figs. 1, 4b).

### Treatment with ECQ enhanced clustering of LDs and accumulation of triacyglycerol (TAG)

Lipid droplets (LDs) are versatile organelles with vital roles in lipid metabolism and energy homeostasis in all eukaryotes. LDs biogenesis occurs at specific sites in the ER, in response to excess amounts of lipids or nutrients, and oxidative stress^44^. In normal conditions, wild-type cells produce small LDs that are loosely circulated in cells (Fig. 4c). After treatment with *Xylaria* extract or ECQ, the wild-type yeast cells showed aggregation of LDs and increased Nile Red fluorescent intensity detection when compared with the untreated cells (Fig. 4c). Nile Red staining and fluorescence microscopy are commonly used to examine cellular neutral lipids, comprising of LDs. This suggested increased accumulation of toxic lipids inside the cells. The Δ*erg6* strain also exhibited LDs aggregation with aberrant Nile Red fluorescent intensity detection when compared to the wild-type strain, suggesting increased accumulation of toxic sterols (Fig. 4b, c). Interestingly, treatment with *Xylaria* extract or ECQ showed strong Nile Red fluorescent intensity of many big clusters or fusion of LDs, suggesting a mobility block of LDs filled with excessive toxic lipid squalene within the Δ*erg6* cells (Fig. 4b,c). The results showed not only significant lipid droplet clustering which arbitrarily is defined as an aggregation of six or more lipid droplets as previously shown in the Δ*erg1* strain with defective in sterol biosynthesis^45^.

Formation of LD cluster agreed well with the repression of *ERG1* expression by ECQ in the wild-type strain (Figs. 2, 4c). The observed fusion of LDs was also found following the striking formation of supersized LDs and clusters of multiple small LDs in the Δ*erg6* strain (Fig. 4c) as shown for the yeast mutant Δ*fld1* lacking a human seipin homolog, implicated in congenital lipodystrophy^46^. Interestingly, previous work demonstrates that LD biogenesis sites at ER subdomains containing Fld1, Nem1, Yft2, and Pex30 define sites for the localization of the TAG-synthase and droplet formation^47^. Consequently, Erg6 and Pet10 bind to newly formed LDs from the cytosolic side. As a result, deletion of *ERG6* caused a devastation in proper LD biogenesis which is critical for cellular function and response to lipotoxicity. Furthermore, the replacement of ergosterol by possibly zymosterol in the plasma membrane of yeast cells^48^ exacerbated the effect of squalene toxicity displayed by the antifungal ECQ, supporting increased sensitivity of in the Δ*erg6* strain, following the ECQ treatment (Figs. 1, 4). Accumulation of free toxic sterol inside the yeast Δ*erg6* strain may exacerbate the effect of ECQ-mediated inhibition of actin cytoskeleton.

Moreover, LDs are cellular organelle involved in storage of metabolic energy in the form of neutral lipids such as sterol esters and triacylglycerol (TAG)^49^. Increased accumulation of lipid droplets was induced by *Xylaria* extract or ECQ treatment (Fig. 4d). Following treatments with the *Xylaria* extract or ECQ, in the wild type cells, TAG content was increased in dose-dependent manner with increasing concentrations of *Xylaria* extract or ECQ from 500 to 1000 µg/ml or 225 to 450 µg/ml, respectively (Fig. 4d). While in the ∆*erg6* strain, high accumulation of TAG occurred after treatment with lower concentrations of *Xylaria* extract or ECQ at 225 or 112 µg/ml, respectively (Fig. 4d). Since increasing concentrations of *Xylaria* extract or ECQ caused dramatic growth inhibition of the ∆*erg6* cells, this resulted in low numbers of cells not enough for TAG detection. Thus, ECQ treatment not only affect sterol composition but also resulted in accumulation of neutral lipid TAG that is correlated with increased numbers and size of LDs (Fig. 4d). It remains to be shown whether ECQ action on actin disruption affects LD mobility.

## Discussion

**Figure 5:**
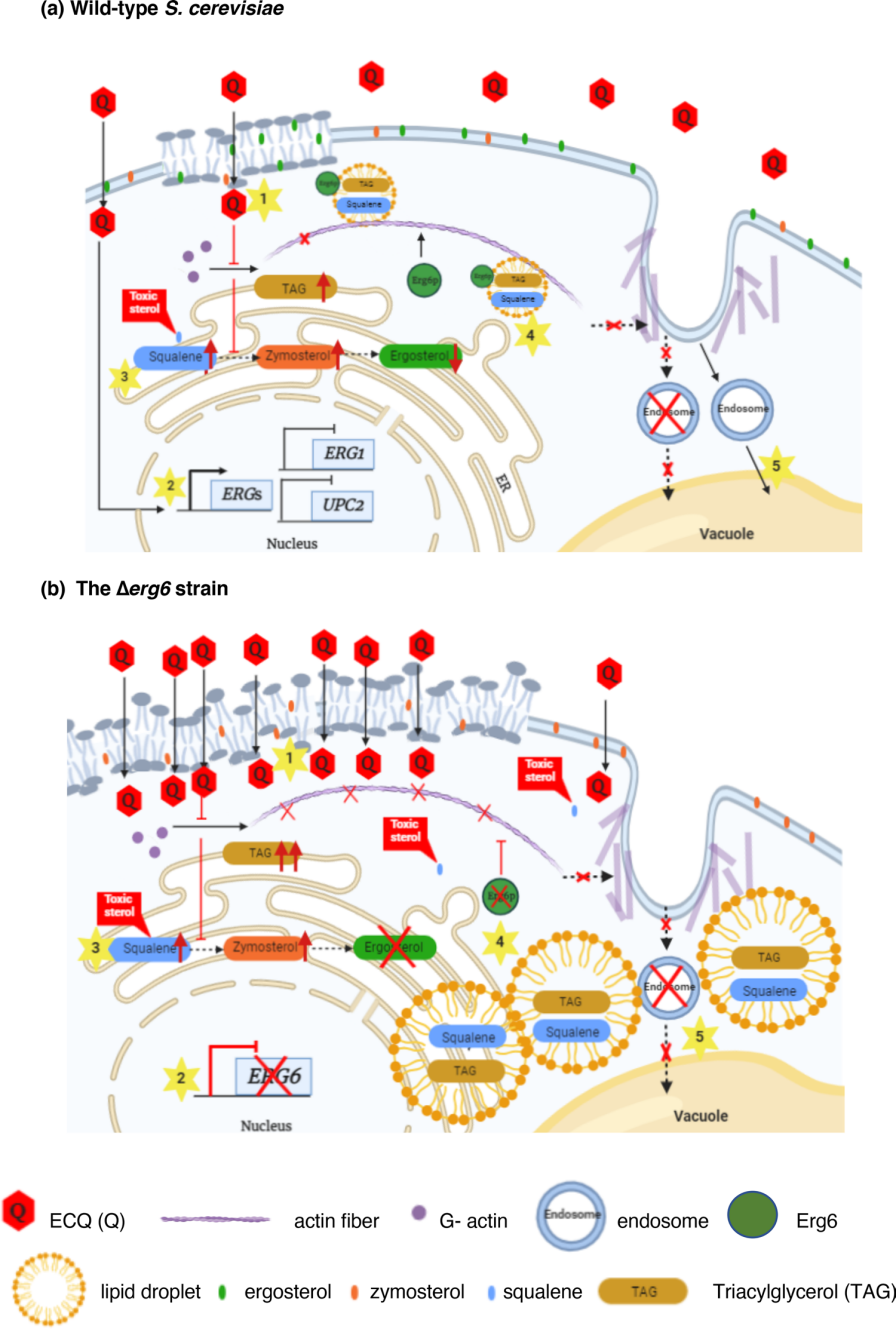
Mode of action of antifungal ECQ in the model yeast *S. cerevisiae.* (**a**) In wild-type strain, once entered the cells (1), ECQ not only disrupted actin cytoskeleton organization but also strongly induced expression of *ERG* genes, including *ERG6,* to compensate for lower ergosterol level (2). ECQ treatment resulted in the alteration of lipid composition (3), leading to increased accumulation of TAG as well as zymosterol and toxic sterol squalene that normally being stored in LDs. LDs formation was induced (4) while some aggregated actin and damaged protein were also removed via endosomes (5) to reduce effect of ECQ cytotoxicity. (**b**) In contrast to the ∆*erg6* strain, membrane permeability was increased (1), allowing penetration of drugs including ECQ. Loss of Erg6 caused pronounced effects on ECQ-mediated inhibition of actin cytoskeleton organization and function (2) and alteration of ergosterol metabolism (3), resulting in elevated levels zymosterol, the defective plasma membrane and abnormal cell morphology. Additionally, despite formation of LDs, they were clustered and non-functional (4), resulting in increased accumulation of toxic squalene. However, newly formed LDs could not be properly destined for vacuolar degradation as a result of ECQ inhibition on actin transport of LDs (5). Overall, ECQ displayed a coupling antifungal mechanism, resulting in abnormal actin cytoskeleton organization and altered sterol and lipid homeostasis. Red arrows indicated a decreased or increased of cellular sterol or TAG levels after treatments with ECQ. Yellow star number 1–5 indicated proposed cellular events during ECQ treatments.

Antimicrobial drug resistance is presently a worldwide important health concern. Using the model yeast *S. cerevisiae*, a role of antifungal 19,20-epoxycytochalasin Q (ECQ) as a novel sterol modulator and its mechanism of actions are summarized (Fig. 5). Noticeably, varied drug concentrations used in each assay are determined according to cell growth/survivability of wild-type and *erg6*Δ strains. The range of ECQ concentrations required for detection of certain cellular events of a variety of experiments, varied between 0 and 1000 µg/ml, is dependent on the strain’s genetic backgrounds. The concentrations used are adjusted, according to the MIC_80_ of drugs or the extract to provide comparable numbers of cells in each experimental tested condition. Alternatively, if possible, results of each assay operated by overlapping concentrations of drugs are shown in parallel. For this reason, the relationships between the observed cellular events shall be considered conditionally. Nevertheless, the data on genetical, metabolic or morphological changes could be discussed when the concentrations of ECQ used were comparable. To summarize, treatment of the wild-type strain with ECQ at 500 µg/ml caused up-regulation of some *ERG*s genes but down-regulation of *ERG1* and *UPC2* genes (Fig. 2). At similar ECQ concentration of 450 μg/ml, in the wild-type strain, disruption of actin organization was slightly observed as shown by aggregated actin fiber and increased accumulation of actin bodies (Fig. 3). Moreover, sterol homeostasis was disrupted as shown by decreased ergosterol content, increased squalene and zymosterol content and increased LDs aggregation with increased accumulation of TAG content (Fig. 4). However, more impaired metabolic and morphological effects were observed in the Δ*erg6* strain at even lower ECQ concentrations used. At 225 μg/ml of ECQ, disruption of actin organization occurred faster as shown by aggregated actin fiber and increased accumulation of actin patches and actin bodies at 2 h (Fig. 3). At this ECQ concentration, cells began to die and, there were not enough cells for recovery of sterol or TAG (data not shown). Thus, a lower ECQ concentration of 112 μg/ml was used. Increased squalene and TAG accumulation as well as increased LDs aggregation were evidently found as compared to the untreated condition (Figs. 3 and 4). More severe morphological effect was observed at 24 h of treatment as shown by disrupted actin structure (Fig. 3). Cell membrane breakage was evidently observed in the wild-type and the Δ*erg6* strains at 800 μg/ml of ECQ treatment (Fig. 3) although this may happen already at lower ECQ concentrations. Overall, results indicated that there was a correlation between genetical, metabolic and morphological changes of cells in response to ECQ treatments (Fig. 5).

Here, some important aspects on molecular mechanisms and cellular responses to ECQ could be drawn. First, several lines of evidence support the requirement of some Erg enzymes including Erg6p, in the adaptive cellular response to ECQ, as shown by the increasing tolerance of these *ERG*-overexpressing strains and altered expression levels upon ECQ treatments (Figs. 1, 2). Interestingly, gene expression analysis of ECQ treated wild-type yeast cells showed down regulation of *UPC2* and *ERG1* genes but not *ECM22* which is opposite to regulation of other *ERG* genes tested (Fig. 2). In sterol-rich and aerobic condition, the basal *ERG* gene expression is mostly maintained by Ecm22p and Hap1p^11^. However, under ergosterol depletion as in the case of ECQ-treated cells, Upc2p is a key regulatory protein to activate the transcription of *ERG* genes and its own expression^11^. This may explain differential regulation observed in the gene expression analysis. Secondly, adaptive cellular response to the actin inhibitors or ECQ may operate to prevent activation of the whole ergosterol biosynthetic pathway or through selective regulation at some specific *ERG* promoters or at the first step of pathway via repression of *UPC2* and *ERG1* expression, respectively, in order to fine-tune lipid composition and function. Such feedback mechanisms may be possible for a following reason. Firstly, *ERG1* is essential under aerobic growth conditions, but *erg1* null mutants are viable when grown under anaerobic conditions in the presence of ergosterol^50^. Treatment with ECQ reduced expression of *ERG1* transcripts by approximately threefold (Fig. 2). Reduced expression of *ERG1* may affect amount of Erg1p or its enzymatic function which is required for proper growth and cell survival under aerobic condition. Thereby, cells may mechanistically respond through ECQ-mediated *ERG1* repression by activating the downstream ergosterol biosynthetic genes to enhance ergosterol production. Also, repression of *ERG1* has been shown to cause abnormal lipid particle morphology and accumulation of elevated levels of squalene^45^. Thus, activation of genes in the latter steps of ergosterol biosynthesis may be required for controlling level of excess intracellular squalene and maintain lipid homeostasis.

Second, this study reveals the ability of natural antifungal ECQ of the medicinal mushroom *Xylaria spp.* to aggregate actin and to alter biosynthesis of key components of fungal plasma membrane TAG and sterol in yeast cells (Figs. 3, 4). Despite, there is less effect on sterol composition in the *erg6*∆ strain. This indicates that changes of sterol in wild-type cells, at high ECQ concentrations, may be quite indirect. Likely, it may be via a non-sterol target for ECQ, and possibly for extract, which has followed on effects on sterol as shown by the accumulation of LDs (Fig. 4). Thirdly, increased formation of LDs clustering is found following ECQ treatment, especially in the absence of Erg6p (Fig. 4). A mechanism of ER-sterol misplacement mediated through actin cytoskeleton on non-vesicular lipid transport through Erg6-assisociated LDs is suspected to occur during the ECQ treatments (Fig. 5). Defective actin depolymerization and disrupted lipid homeostasis are evident in the Δ*erg6* strain (Figs. 3, 4) which likely increase ECQ and drug uptake which is account for the strain’s susceptibility (Fig. 1). In fact, the null mutation of *ERG6* renders the yeast strain increased sensitivity to various drugs^51^.

In yeast and fungi, sterols are synthesized in the endoplasmic reticulum (ER) and then transported to other cell membranes via non-vesicular lipid transport^52^. Excess free sterols in the ER are cytotoxic; however, they could be detoxified by neutral lipid synthetic enzymes and stored in the form of LDs^53,54^. Despite LDs being known for having a role in the storage of neutral lipids, they also play vital roles in lipid metabolism and homeostasis with important links to the dysfunction of LD-associated diseases in humans^55^. Furthermore, Erg6p is involved in the formation of lipid organelles or LDs in the neutral form of lipids^53,56^. An interatomic map of protein–protein contacts of LDs with mitochondria and peroxisomes also reveals the involvement of LD proteins Erg6p and Pet10p in 75% of interactions detected, suggesting a key role of Erg6p in coordinating cellular signalling and response through formation of LDs^57^. The protein–protein interaction of Erg6p and mitochondrial NADH-cytochrome b5 reductase Mcr1p that is involved in outer membrane sorting has been reported^57^. This interaction suggests a connection between Erg6p and Mcr1p mediation of LD-assisted transfer of substrates in the different sub-mitochondria^57^.

Treatment with a squalene monooxygenase inhibitor terbinafine results in increasing sensitivity of LD-less mutants^43^. The accumulation of the toxic lipid species squalene in yeasts occurs in LD-less mutants, as the excess squalene is normally stored in LDs^42,43^. Increasing ECQ concentrations mediate the accumulation of squalene which increases lethality in the LD-defective Δ*erg6* strain but not in the wild-type strain with proper formation and function of LDs (Fig. 4). Measurement of intracellular squalene in LDs fraction could be further investigated. The defective squalene storage may result in lipotoxicity in yeast cells, leading to increased sensitivity in the mutant *erg6*Δ strain (Fig. 1). A number of reports described elevated of LDs in tumor tissues and, a recent study shows its association with drug resistance in lung cancer cells in which ER stress and apoptosis are shown to be suppressed^58^. Here, ECQ study also supports a connection between the role of highly dynamic organelle LDs and enhanced resistance to drugs.

Moreover, beside LDs, Erg6p is localized at various organelles namely the plasma membrane-associated endoplasmic reticulum and the mitochondrial outer membrane^59–61^. Its versatile setting suggested different and important roles of Erg6p in maintaining lipid and cell homeostasis, especially during cellular response to cytotoxic agents. As mentioned, Erg6p function is highly complex, involving over 1050 total interactions for 615 unique genes according to the SGD database. In attempt to explain the involvement of Erg6p during the ECQ treatment in the wild-type cells, increased LDs clustering where Erg6p likely locates suggests that Erg6p may move to various organelles according to LD trafficking route and finally get degraded via lipophagic process (Fig. 5). In contrast, in the ECQ-treated Δ*erg6* strain, LDs are likely trapped and tethered to the ERs due to ECQ action in targeting actin (Figs. 3, 5).

The effect of ECQ is more evident in the sensitive Δ*erg6* strain due to increased plasma membrane permeability (Figs. 1, 4). It is intriguing question how Erg6p might be functionally inhibited by ECQ? Our results support ECQ action on actin aggregation and a link between actin-mediated mobility of LDs. Cells may respond to ECQ by inducing the *ERG6* and additional *ERG* gene expression (Fig. 2) to restore balance of lipids and maintain numerous critical functions of sterol in yeast cells. Depletion of Erg6p or deletion of *ERG6* results in depletion of ergosterol, therefore compromising the function of fungal drug efflux transporters including *Candida* Cdr1p and *S. cerevisiae* multi-drug transporter Pdr5p^28,54^. Thus, ECQ treatment exacerbates the effect of *ERG6* deletion on lipid and sterol composition and content (Fig. 4), compromising the function of key drug efflux pumps or membrane associated proteins.

Despite some unanswered questions, several studies support Erg6p connecting function in LDs mobility. For example, the antifungal drug miconazole that targets Erg11p has also been shown to induce changes in actin cytoskeleton and activates several genes, involved in membrane trafficking, endocytosis and regulation of actin cytoskeleton, required for miconazole tolerance^62^. Due to a tight regulation of actin cytoskeleton organization and lipid homeostasis, particularly on formation and distribution of LDs which Erg6p plays an essential role for protein recycling as well as proper intracellular and surface cell delivery^63^. Yeast membrane lipid imbalance has been shown to cause trafficking defects between different organelles^64^. The membrane lipid mutant cells have been found to display lower levels of actin cables, suggesting that the actin cytoskeleton is disrupted upon membrane lipid imbalance. Recent study also indicates roles for LDs biogenesis and microlipophagy in adaptation to phosphatidylcholine lipid imbalance in yeast^65^. They report that lipid imbalance triggers several defects including, mitochondria and ER morphology, localization, motility and inheritance of mitochondria. Yeast adapts through LDs biogenesis at ER aggregates in accompanied with increased level of TAG and relevant TAG biosynthetic enzymes. Our analysis of TAG and sterol content in ECQ treated cells also supports such adaptation (Fig. 4).

Finally, stress-induced LD enriched with ubiquitinated proteins are then destined for vacuolar degradation via a process resembles microautophagy. Additionally, another study also shows that the endosomal sorting complex that plays role in transport, membrane sealing, trafficking and autophagy negatively regulates degradation of Erg6p-associated protein lipid droplets under glucose restricted conditions^66^. Overexpression or inactivation of enzymes of ergosterol, phospholipid or sphingolipid biosynthesis affects cellular trafficking. Membrane fusion is also bypassed by increased sterols as shown by overexpression of *ERG6*, which promotes actin remodeling as part the membrane fusion mechanism^67^. Additionally, high accumulation of zymosterol in the Δ*erg6* strain is also observed (Fig. 4) as previously reported by another study^39^ because Erg6p functions to convert zymosterol to fecosterol. Since zymosterol is a toxic sterol that interferes with the integrity and function of yeast cell membranes^48^, an increased level of zymosterol is partly responsible for the growth defects of the ∆*erg6* strain following ECQ treatment (Fig. 1, 4). In fact, zymosterol that circulates within the cells is the key precursor of ergosterol in fungi and of cholesterol in humans. In human fibroblasts, zymosterol is converted to cholesterol solely in the rough ER. Little or no zymosterol or cholesterol accumulates in the ER in vivo, because newly synthesized zymosterol moves to the plasma membrane without a detectable lag or about twice as fast as cholesterol^60^. Since ECQ treatment results in replacement of membrane sterol from ergosterol to zymosterol (Fig. 4), combinatorial treatment using antifungal ECQ and an inhibitor of the fungal-specific enzyme Erg6p may enhance drug sensitivity, offering a potential therapeutic approach in targeting pathogenic yeasts and fungi. Interestingly, Erg6p is the one of enzymes in ergosterol biosynthesis that are not involved in the cholesterol biosynthetic pathway. As mentioned Erg6p is responsible for the conversion of zymosterol to fecosterol and it is currently a promising and critical target for new antifungal drugs. Some inhibitors of Erg6p have been reported, such as azasterols; however, their use is limited due to the undesirable inhibition of the human enzyme 24-sterol reductase, which causes adverse toxic effects^68,69^.

Indeed, a correlation between the organization of the actin cytoskeleton and sterol synthesis has been also implicated in cell proliferation. To explore the benefits of ECQ, our previous work shows that ECQ enhances antifungal action of azole drugs and lowers dosage used of ECQ by approximately 10–50 times when combined with some azoles^23^. Although most studies on cytochalasins are explored in mammalian cell lines, effect of cytochalasin B and D at concentrations of 5 and 1 µg/ml is reported to complete actin disruption^33^. Much lower concentrations required as compared to yeast cells. Effects of cytochalasans B, D and E and novel phenochalasins are also found on cytosolic lipid droplet formation and neutral lipid synthesis as well as their ability to inhibit cholesteryl ester (CE) synthesis in mouse peritoneal macrophages with IC50 values ranging between 0.1 and 20 µM^70^. Additionally, cytochalasins effects on sterol metabolism and plasma membrane integrity are shown. For examples, intracellular uptake and esterification of cholesterol are blocked by cytochalasin D in human monocyte-derived macropages^71^. The actin cytoskeleton is important for the stimulation of cholesterol esterification by atherogenic lipoproteins in macrophages. Cytochalasin D treatment of macrophages also inhibits the ability of acetyl-low density lipoprotein to stimulate cholesterol esterification^72^. Further, inhibition of plasma membrane cholesterol internalization by Cytochalasin D inhibitor causes dose-dependent inhibition of steroid hormone synthesis in the MA-10 cells^73^. Therefore, appropriated dose of cytochalasins used in therapeutic treatments shall be clinically investigated. Optimization of ECQ production will be required for future experiments and commercially availability. Media optimization has been reported for improving ECQ production which provides approximately 4.4-fold increase of yield^74^. Work is in progress to increase production of ECQ.

Importantly, some studies report benefits of cytochalasins in cancer treatments including increased the life expectancy and prolonged survival of mice challenged with leukemias^75^. Thus, in addition to their fungicidal activity, cytochalasins have unique antineoplastic activity with promising potential as a novel chemotherapeutic agent^76^. Interestingly, squalene epoxidase is the not only target of fungicides but recently also gains increasing interest as a new target of anti-cancer drugs^77^. Cancer-associated small integral membrane open reading frame 1 (CASIMO1) is a known oncogene that is overexpressed predominantly in breast cancer patient samples. CASIMO1 contributes to the proper formation of the actin filament network and interacts squalene epoxidase, a major enzyme in cholesterol synthesis, of the mevalonate (MVA) pathway^78^. Recently, another study also shows that the cholesterol-independent feedback mechanism of sterol regulatory element-binding protein 1 (SREBP1) regulation is mediated by isoprenoids in regulating cell homoeostasis^79^. Our study in the model yeast *S. cerevisiae* and other recent findings in mouse and *Drosophila* models^80–83^ have unveiled a novel role of functional metabolites with potential implications in human health and disease.

In conclusion, the effects of the cytochalasin ECQ to disrupt actin depolymerization, a process critically affect LDs mobility on cytoskeleton network, and alter sterol metabolism are shown. ECQ-mediated disruption of actin function, results in gigantic formation of LDs clusters especially in the Δ*erg6* mutant since LDs are transported directly along actin filaments^84^. Given a vital role of LDs on various life activities, including membrane homeostasis, investigation on membrane integrity or abnormal membrane microdomains is warranted.

## Materials and methods

### *Xylaria* culture and extraction

*Xylaria* sp. BCC1067 was obtained from the BIOTECH Culture Collection (BCC culture 6,200,032,292); National and Technology Development Agency, Bangkok, Thailand). Cultivation of *Xylaria* sp. BCC 1067 was done according to the method by Phonghanpot et al.^85^, with some modifications, as described by Somboon et al.^22^ *Xylaria* sp. BCC 1067 was grown on solid media containing 1.5% (w/v) malt extract broth (MEB, OXOID, Oxoid Ltd., UK) and 2% (w/v) agar (HIMEDIA, India) then transferred to fresh liquid MEB and allowed to grow for 25 days at 25 °C without shaking. After, the culture was separated and the cultural media fraction was then extracted with ethyl acetate (QREC, New Zealand) and dried under rotary evaporation. The dried crude extract was freshly dissolved with methanol prior to use.

### Isolation of 19,20-epoxycytochalasin Q from the *Xylaria* sp. BCC 1067 extract

The isolation of bioactive compounds was described by Elias et al*.*, with modifications^86^. One gram of crude *Xylaria* extract obtained from the cultural media fraction was subjected to silica gel column chromatography ($\varnothing$ 2.5 cm × 25 cm). The column was eluted with CH_2_Cl_2_/EtOAC/MeOH and yielded the following 5 fractions (F1–F5): (ratio of CH_2_Cl_2_–EtOAc (5:95, 0:100 v/v) 100 ml, EtOAc 100 ml and ratio of EtOAc –MeOH (90:10, v/v) 50 ml). The pure ECQ compound was found in fraction F3, and F3 was then isolated further. The fraction F3 was subjected to silica gel column chromatography ($\varnothing$ 2 cm × 25 cm) using gradient CH_2_Cl_2_–EtOAc. The fraction F3 was eluted with a gradient of solvent, as described, to give 9 fractions (S1–S9). The ECQ was found in fraction S5 that is further isolated by silica gel column chromatography ($\varnothing$ 1.5 cm × 23 cm) using a gradient of CH_2_Cl_2_–EtOAc. The fraction S5 was eluted and, the fraction S5-1 was identified by High Performance Liquid Chromatography (HPLC) and determined by nuclear magnetic resonance (NMR) as 19,20-epoxycytochalasin Q.

### Susceptibility assays of yeast deletion and *ERG* overexpression strains

The susceptibility of *S. cerevisiae* yeast deletion strains (Table 1) was testes against fluconazole, *Xylaria* sp. BCC 1067 extract, or ECQ using the microdilution reference method from the National Committee for Clinical Laboratory Standards^87^, with modifications according to Somboon et al.^22^. Yeast strains were treated with *Xylaria* extract or ECQ or fluconazole in 96-well plate and incubated at 150 rpm and 30 °C for 24 h. OD_600_ of cells were measured by using an automated microplate reader (M965 + ; METERTECH, Taipei, Taiwan). Normalized growth was calculated from normalized of OD_600_ of treated cells compared to untreated cells. Then, 3 µl of cells were spotted on YPD agar plates and incubated for 48 h to observe cell survival. The standard culture media YPD (HIMEDIA, India) was utilized for MIC and MFC determinations. In addition, the susceptibility of yeast strains OVER-*ERG1*, OVER-*ERG6,* OVER-*ERG11* and BY4742 + empty vector was investigated against ECQ using the microdilution reference method from the National Committee for Clinical Laboratory Standards^87^, with modifications. Yeast strains were cultured overnight under YPDG and adjusted to OD_600_ of 0.001 and regrown in YPG to induce expression of gene under *GAL1* promotor. In 96-well plates, yeast cells were treated with different concentrations of ECQ and incubated at 30 °C with shaking at 150 rpm for 36 h. OD_600_ of cells were measured by using an automated microplate reader (M965 + ; METERTECH, Taipei, Taiwan). Normalized growth was calculated from normalized of OD_600_ of ECQ-treated cells compared to untreated cells. Then, cells from micro-dilution assays were directly spotted (10^0^ dilution) or 1000 times diluted (10^–3^ dilution) on YPD plates to examine the survivability.

**Table 1:** Genotypes *S. cerevisiae* wild-type and deletion mutant strains used in this study.

Strain	Genotype	Reference
BY4742	MATα his3Δ1 leu2Δ0 lys2Δ0 ura3Δ0	Open Biosystems
Δ*upc2*	BY4742 (*MATα his3Δ1 leu2Δ0 lys2Δ0ura3Δ0*) *Δupc2::kanMX4*	Open Biosystems
Δ*hmg1*	BY4742 (*MATα his3Δ1 leu2Δ0 lys2Δ0ura3Δ0*) *Δhmg1::kanMX4*	Open Biosystems
Δ*sut1*	BY4742 (MATα his3Δ1 leu2Δ0 lys2Δ0ura3Δ0) Δ*sut1*::kanMX4	Open Biosystems
Δ*erg4*	BY4742 (*MATα his3Δ1 leu2Δ0 lys2Δ0ura3Δ0*) *Δerg4::kanMX4*	Open Biosystems
Δ*erg5*	BY4742 (*MATα his3Δ1 leu2Δ0 lys2Δ0ura3Δ0*) *Δerg5::kanMX4*	Open Biosystems
Δ*erg6*	BY4742 (*MATα his3Δ1 leu2Δ0 lys2Δ0ura3Δ0*) *Δerg6::kanMX4*	Open Biosystems
Δ*erg28*	BY4742 (*MATα his3Δ1 leu2Δ0 lys2Δ0ura3Δ0*) *Δerg28::kanMX4*	Open Biosystems

### Gene induction and quantitative real-time polymerase chain reaction (qRT-PCR)

The *S. cerevisiae* wild-type strain BY4742 was cultured in YPD at 30 °C with shaking overnight. The yeast cells were measured and adjusted at an optical density (OD_600_) of 0.1 for the starter cell. The culture was incubated until obtaining an OD_600_ of about 0.5. Then, 4 μg/ml of ketoconazole or 500 μg/ml of *Xylaria* extract or 500 μg/ml of ECQ was applied to cell culture for gene induction for 2 h. Total RNAs were extracted as described by Schmitt et al.^88^. RNA was dissolved by DEPC and purified using the RNeasy Mini Kit (QIAGEN, Hilden, Germany). The purified RNA was used to synthesize cDNA by using the qPCRBIO cDNA synthesis kit (PCRBIOSYSTEMS, UK). The qRT-PCR assays were performed using a Real-Time PCR Detection System with a software for analysis. The reaction mixtures contained Universal qPCR Master Mix (NEB). Gene-specific oligonucleotides were used are shown (Table 2). The relative expression data were analyzed using the 2^–ΔΔCt^ method.

**Table 2:** Primers used in the real-time RT-PCR and plasmid construction.

Primer	Sequence
*UPC2-F*	CTCCTACGATCAAGAAGGAGC
*UPC2-R*	GAGATTGCTGCTGCACTTG
*ECM22-F*	CATCAGCGGTCCACGATA
*ECM22-R*	TGTTACCGCACCTATTAGCG
*ERG1-F*	ATTCCATACCCTTACAAGGCC G
*ERG1-R*	CGTGCATAGGAGCAGGATTC
*ERG2-F*	CCACTGAAGACCTGTTACAGG
*ERG2-R*	ACCTGTGTGCCCTTCAGTA
*ERG3-F*	AGAAGGTTCTACGGGCAGG
*ERG3-R*	GGATAGCACTGACTGCCAA
*ERG4-F*	CACGGTAAGGTTGCCCTAC
*ERG4-R*	ACATGCGTTCGCGTACAA
*ERG5-F*	CGTTGTCGATGTTGCTGTGA
*ERG5-R*	CTTCATGGCCATGTCTGC A
*ERG6-F*	CCCAGCAAGAGAGATTGCA
*ERG6-R*	AGGTCTTCGCTAACGAGGAC
*ERG9-F*	GACGATATGTCCATCGAACAC G
*ERG9-R*	GACAGTACACGTCGTAGTCG
*ERG11-F*	GACCGTCCACCTCTAGTGT
*ERG11-R*	CGTAAGCAGCTTCTGCTGA
*ERG1OVER_F1*	CACTTAGGATCCATGTCTGCTG
*ERG1OVER_R1*	CTGCCGCTCGAGTTAACCAATC
*ERG6OVER_F*	CGAGGGTACATGGCGGATCCATGAGTGAAACAGAATTGAG
*ERG1OVER_R*	CTAGCAACGATCGGCTCGAGTTATTGAGTTGCTTCTTGGG
*ERG11OVER_F*	GGCATTAGCGTGCGGATCCATGTCTGCTACCAAGTCAATC
*ERG11OVER_R*	GTCGGATGCGGCTCGAGTTAGATCTTTTGTTCTGGATTTC

### Quantification of sterols via gas chromatography-mass spectrometry (GC–MS)

Quantification of sterol components was described by Li et al.^89^. The yeast strains (2 × 10^5^ cells/ml in YPD medium), treated or untreated with the *Xylaria* extract or ECQ. The wild type cells were treated with 1000 or 225 µg/ml of *Xylaria* extract or ECQ, respectively. While the ∆*erg6* cells were treated with 225 or 112 µg/ml of *Xylaria* extract or ECQ, respectively. Concentration at MIC/4 was used to treat ∆*erg6* strain with the *Xylaria* extract or ECQ and wild type strain with ECQ. For wild-type cells, 1000 µg/ml of *Xylaria* extract was used for treatment because could not find MIC from susceptibility test. For GC–MS, cells were grown for 24 h at 30 °C with shaking. Cells were then collected and resuspended in 40% alcoholic KOH. The mixture was saponified by heating at 85 °C for 1 h and allowed to cool to room temperature. The sterol was then extracted in petroleum ether and vaporized and analyzed by GC–MS (AGILENT 7890B). The gas chromatograph was equipped with a 5% phenyl and 95% dimethylpolysiloxane column (length, 30 m; inner diameter, 0.25 mm; film thickness, 0.25 µm). The settings were as follows: initial GC temperature of 120 °C for 4 min, 290 °C for 3 min with gradient of 15 °C/min; detector temperature, 340 °C; sample injection temperature, 320 °C; carrier gas, nitrogen gas; flow rate, 1 ml/min. Sterols were identified from their retention times and specific mass spectrometric patterns.

### Actin staining and fluorescence microscopy

The actin staining method was described by Gabriel et al.^90^, with modifications. Wild-type *S. cerevisiae* and the ∆*erg6* strains were cultured in YPD at 30 °C with shaking overnight. The optical density (OD_600_) of the cell culture was measured and adjusted to 0.1 as a starting OD, and cells were incubated until obtaining an OD_600_ of 0.6. Then, wild-type *S. cerevisiae* cells were treated with 1000 or 450 µg/ml of the *Xylaria* extract or ECQ, respectively, while the ∆*erg6* cells were treated with 450 or 225 µg/ml of the *Xylaria* extract or ECQ, respectively. Then, the cells were collected at various time points of 0, 2, 4, 6, 12, and 24 h. Next, the cells were washed twice with Phosphate-buffered saline (PBS) buffer, incubated with 3.7% formaldehyde for 1 h, stained with phalloidin-FITC labelled for 1 h, and then washed twice with PBS buffer. The actin of the cells was examined with a confocal laser scanning microscope (FV10i-DOC).

### Nile Red staining

The Nile Red staining method described by Rostron et al.^91^ was used with some modification. The yeast strains (2 × 10^5^ cells/ml in YPD medium), treated or untreated with the *Xylaria* extract or ECQ, were grown for 24 h at 30 °C with shaking. After that, 250 μL of cells were transferred into sterile 1.5 mL tubes to be stained. 25 μL of freshly prepared DMSO:PBS (1:1) and 5 μg/mL Nile Red in acetone were added, and the cells were incubated in the dark for 5 min at room temperature. The cells were washed twice with PBS and resuspended in 100 μL of 10% (v/v) formalin and fixed for 15 min in the dark at room temperature. The cells were washed twice with PBS and proceeded to imaging using a ZEISS Apotome.2 fluorescent microscope (ZEISS, Germany).

### Quantification of TAG

Quantification of TAG was investigated as previously described by Garaiová et al.^92^ with some modification. The yeast cells (2 × 10^5^ cells/ml in YPD medium), treated or untreated with the *Xylaria* extract or ECQ were collected and washed twice with DI water. Cells were disrupted by vortexing with glass beads and extracted by hot methanol (30 min at 65 °C). Then, cells were further incubated in chloroform–methanol (3:1) at room temperature. The organic phase containing lipids was collected and evaporated under N_2_ stream. Dry lipid residue was dissolved in toluene and investigated TAG content by High Performance Liquid Chromatography-Evaporative Light Scattering Detector (HPLC-ELSD).

### Plasmid construction

To overexpress of *ERG1*, *ERG6* and *ERG11* gene in *S. cerevisiae* BY4742 (GenBank JRIR00000000). The pYES_*ERG1*, pYES_*ERG6* and pYES_*ERG11* plasmids were constructed in this study. The pYES2 plasmid was used as a vector which is the expression plasmid under *GAL1* promotor in yeast expression system. In part of gene insert, the *ERG1*, *ERG6* or *ERG 11* genes were amplified from genomic DNA of *S. cerevisiae* by using High-Fidelity DNA Polymerase (NEW ENGLAND BIOLABS) and used primer that containing the sequence of restriction enzyme (BamHI and XhoI) (Table 2). The amplification condition was carried out as follows; 98 °C for 30 s, and the cycle of denaturation at 98 °C for 10 s, annealing at 45–50 °C for 30–60 s, and extension at 72 °C for 1–2 min. The PCR product of individually gene was then digested with BamHI and XhoI, at 5′ and 3′ sites respectively, and ligated into the BamHI and XhoI sites of pYES2 to yield the plasmid pYES_ERG1, pYES_ERG6 and pYES_ERG11. Transformation was performed following by heat shock method^93^. The transformants were selected on LB agar plate containing 100 µg/ml of ampicillin and verified by restriction enzyme digestion and DNA sequencing. The pYES_*ERG1*, pYES_*ERG6* and pYES_*ERG11* constructed plasmids were individually transformed into *S. cerevisiae* BY4742 by LiAc/SS carrier DNA/PEG method^94^. The transformants were selected on synthetic defined (SD) yeast medium (without Uracil). The sequencing was performed and the expression was checked via qRT-PCR.

### Statistical analysis

The results were presented as mean ± SD and statistical analysis was performed by using one-way.

ANOVA, followed by Tukey’s pairwise comparison by the SPSS Statistics 27.0 software (IBM, NY, USA). A *p* value of < 0.05 was considered as significantly different. At least three independent experiments were performed at least in triplicates.

## Supplementary Information

Supplementary Figure.
