# Supplementary material for: Actin cytoskeletal inhibitor 19,20-epoxycytochalasin Q sensitizes yeast cells lacking ERG6 through actin-targeting and secondarily through disruption of lipid homeostasis
Source: Sci Rep. 2021 Apr 8;11:7779. doi: 10.1038/s41598-021-87342-4 (PMC8032726; doi:10.1038/s41598-021-87342-4)
Supplement: Supplementary file 1 — Supplementary Figure. [file 41598_2021_87342_MOESM1_ESM.pdf]

Actin cytoskeletal inhibitor 19,20-epoxycytochalasin Q modulates disruption of lipid sterol homeostasis in *S. cerevisiae*

Kwanrutai Watchaputi[1], Pichayada Somboon [2], Nipatthra Phromma-in[1], Khanok Ratanakhanokchai[1] and Nitnipa Soontorngun[1,*]

(a)

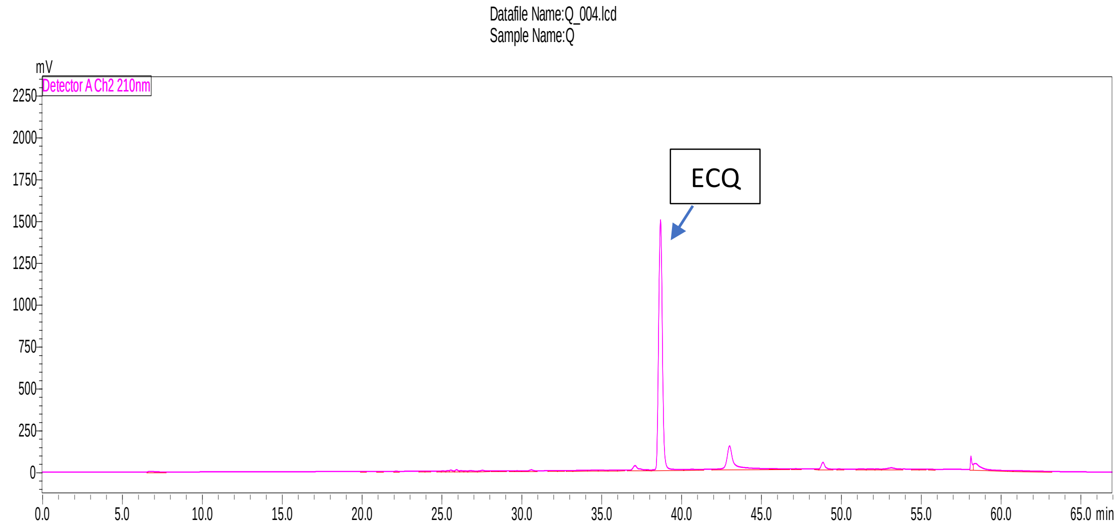

(b)

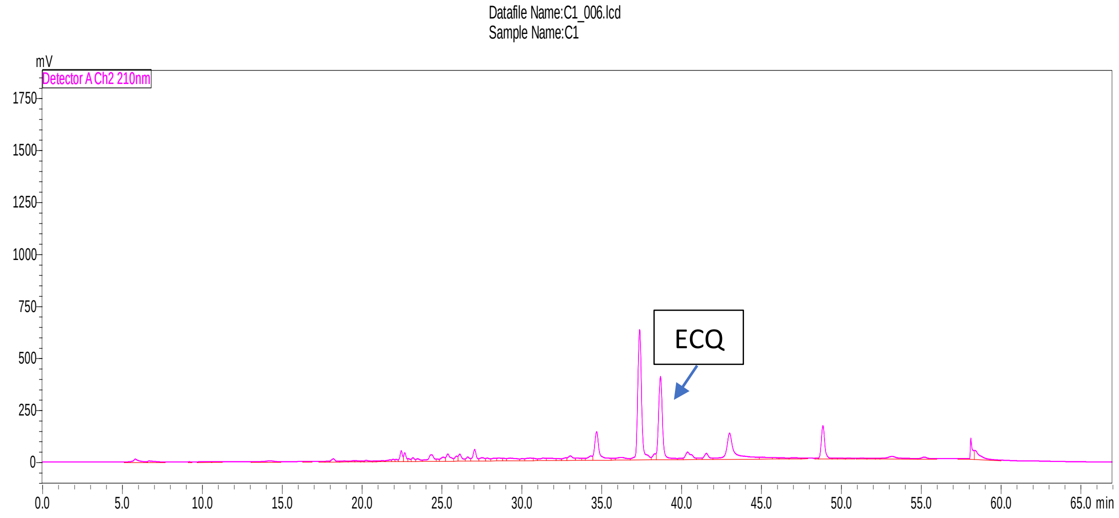

Supplementary Figure 1. HPLC profile of (a) ECQ and (b) the *Xylaria* extract containing ECQ of 27.24%±2.9% in a total extract at retention time at 38.7.
